# The association between blood metals and cardiovascular diseases: findings from National Health and Nutrition Examination Survey 2011–2020

**DOI:** 10.3389/fcvm.2024.1479665

**Published:** 2024-12-18

**Authors:** Bin Li, Haiyue Liu, Devrakshita Mishra, Zhen Yuan, Yizhi Zhang, Longzhen Zhang, Yanshu Huang, Ye Zhang, Ju Lin, Jianyou Chen, Zuheng Liu

**Affiliations:** ^1^ Department of Cardiology, Xiamen Chang Gung Hospital, School of Medicine, Huaqiao University, Xiamen, China; ^2^ Xiamen Key Laboratory of Genetic Testing, Department of Laboratory Medicine, The First Affiliated Hospital of Xiamen University, School of Medicine, Xiamen University, Xiamen, China; ^3^ Department of Clinical Medicine, School of Medicine, Xiamen University, Xiamen, China; ^4^ Department of Cardiology, Xiamen Key Laboratory of Cardiac Electrophysiology, Xiamen Institute of Cardiovascular Diseases, The First Affiliated Hospital of Xiamen University, School of Medicine, Xiamen University, Xiamen, China

**Keywords:** cardiovascular diseases, blood metals, NHANES, lead, cadmium

## Abstract

**Objectives:**

Previous studies have examined the relationship between cardiovascular diseases (CVDs) and blood metal levels. However, fewer studies have investigated the role of the combinations of blood metals on CVDs. In the current study, our aim is to explore the roles of specific blood metals and further develop a model to differentiate between healthy participants and CVD patients using database from the National Health and Nutrition Examination Survey (NHANES).

**Methods:**

Data from the National Health and Nutrition Examination Survey (NHANES) from 2011 to 2020 were collected and utilized in the present study. Demographic characteristics and examination results were gathered and analyzed to compare CVD and non-CVD participants. Logistic regression and random forest analyses were employed to determine the odds ratios and the effects of various blood metals on CVDs.

**Results:**

A total of 23,448 participants were included and analyzed. Participants were divided into CVD (*n* = 2,676, 11.41%) and Non-CVD (*N* = 20,772, 88.59%) groups. A significant difference in the increased odds ratio of CVDs and higher blood Lead levels was found in the logistic analysis [OR (95% CI) = 13.545 (8.470–21.662) *P* < 0.001]. Although this significance blunted in the adjusted model, blood lead levels could be identified as the most important score through the random forest model in distinguishing cardiovascular diseases. In addition, the odds ratio of CVDs in logistic regression was 1.029 (95% CI: 1.022–1.035) for participants with higher blood cadmium levels (*p* < 0.001). The odds ratio increased [OR (95% CI) = 1.041 (95% CI: 1.032–1.049) *P* < 0.001] after the necessary adjustments were made for the gender, age, BMI, race and education background. In addition, blood selenium seems to be a protective factor of CVDs as the odds ratios were 0.650 and 0.786 in the crude and adjusted models, respectively. Additionally, the AUC was 0.91 in the predivtive model made by using the data of clinical indices and blood metals.

**Conclusions:**

In summary, blood metals may play an important role in the onset and progression of CVDs, and they can be used to develop a predictive model for CVDs, which might be beneficial for the identification and early diagnosis of CVDs.

## Graphical Abstract

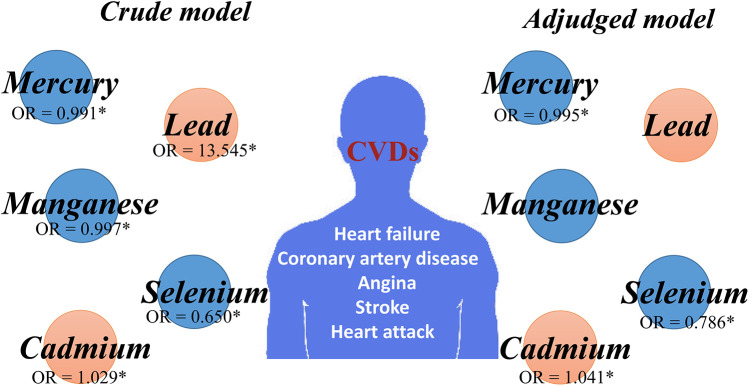

## Introduction

Cardiovascular diseases (CVDs) are prevalent globally and pose a serious threat to human health and life (1). Traditional risk factors for CVDs include disorders of glucose or lipid metabolism, such as diabetes mellitus, hyperlipemia, obesity, and metabolic syndrome, as well as unhealthy living habits like smoking, a high salt diet, and a sedentary lifestyle (2, 3). However, despite the identification and verification of these various risk factors, they do not fully account for the risk of developing CVDs. Environmental pollution, particularly air pollution and exposure to certain heavy metals, has been well-documented as being closely linked to the onset and progression of CVDs (4). These heavy metals can interfere with oxidative stress reactions and induce chronic inflammation, thereby adversely affecting cardiovascular health (5, 6). As a result, the adverse effects of heavy metals have been observed in various epidemiological studies and have garnered significant public concerns in recent years.

Generally, heavy metals refer to metals which have a density at least four times greater than that of water, including copper, lead, zinc, tin, nickel, cobalt, antimony, mercury, cadmium, and bismuth (7). Heavy metals are non-biodegradable and can be enriched and amplified under the food chain, eventually entering and accumulating in the human body. When heavy metals interact with the proteins and inactive enzymes in our body, they can contribute to chronic poisoning.

Although emerging evidence indicates a close relationship between CVDs and metals (5, 8), there are still few studies investigating the role of the mixtures of blood and metals in CVDs. Given these previous findings, data from the National Health and Nutrition Examination Survey (NHANES) 2011–2020 were collected and analyzed to explore the association between various blood metals and CVDs. We aim to explore the effect of heavy metal co-exposures on the risk of CVDs and further identify the crucial blood metals that are correlated with CVDs.

## Materials and methods

### Study sample

The NHANES data were obtained and used for the present study. We conducted a cross-sectional study of participants within the time period of 2011–2020. NHANES is an investigation program of the National Center for Health Statistics (NCHS) in the USA, which collects health and nutritional information from the American population through interviews and laboratory or medical examinations on participants. The survey is conducted biennially and encompasses the collection of demographic, dietary, examination, and laboratory data. NHANES employs a multi-stage sampling design to represent the U.S. civilian population. It first selects primary units (counties), then divides them, lists the households, and randomly selects from these lists. Subsequently, individuals are chosen based on age, sex, and race/ethnicity. NHANES utilizes stratified sampling and oversampling techniques to enhance the accuracy of subgroup estimates. The weight assigned to each individual reflects their representation in the population, thereby ensuring a nationally representative sample for research purposes. NHANES is a publicly available database, the above-mentioned information can be accessed publicly via the following URL: https://www.cdc.gov/nchs/nhanes/.

In our study, data from NHANES 2011–2020 were aggregated to augment the sample sizes. We recruited individuals with complete blood metal information and cardiovascular disease (CVD) information. CVDs are include heart failure, coronary artery diseases such as angina, myocardial infarction or stroke. The verification of heart failure, coronary artery disease, angina, stroke, or heart attack is based on the questionnaire of MCQ160B, MCQ160C, MCQ160D, MCQ160F, and MCQ160E, respectively. Hypertension was verified by the questionnaire of BPQ020, which asked “Ever told you had high blood pressure”. Diabetes was verified by the questionnaire of DIQ010, which asking “Doctor mentioned you have diabetes”. Demographic information and characteristics, including age, gender, BMI, race, education, and current smoking status, were obtained through interviews. The indicators for blood heavy metal levels are sourced from the NHANES database, and their respective identifiers are LBDBPBSI: Blood lead (umol/L), LBDBCDSI: Blood cadmium (nmol/L), LBDTHGSI: Blood mercury, total (nmol/L), LBDBSESI: Blood selenium (umol/L) and LBDBMNSI: Blood manganese (umol/L). Meanwhile, we can retrieve their specific indicator description information (including the years of recording) from the search interface at https://wwwn.cdc.gov/nchs/nhanes/search/default.aspx. Data for these indicators are available for the years 2011–2020. Data from before 2011 have not been collected into our group due to the absence of test results for these indicators.

Detailed instructions regarding specimen collection and processing can be found in the NHANES Laboratory/Medical Technologists Procedures Manual (LPM). Vials are stored under appropriate frozen conditions (–30°C) until they are shipped to the National Center for Environmental Health for testing. Whole blood concentrations of lead (Pb), cadmium (Cd), total mercury (THg), manganese (Mn), and selenium (Se) are determined using inductively coupled plasma mass spectrometry (ICP-MS). This multi-element analytical technique is based on quadrupole ICP-MS technology. Then, we also analyzed the relationship between cardiovascular mortality and blood heavy metals from 2011 to 2018, using the accessible mortality data from that period. In the current analysis, 68,488 participants from NHANES were enrolled. This present study is a secondary data analysis which lacks personal identifiers and does not require institutional reviewing. However, all procedures conducted in the NHANES were approved by the National Center for Health Statistics Research Ethics Review Board, and all participants were provided with a written informed consent.

### Statistical analysis

The data were processed using *R* or SPSS 20.0. Student's *t*-test analysis or the Mann–Whitney *U* test was used for comparing continuous variables, while the chi-square test was used to compare the composition ratios of each group. The Levene test was performed to assess the homogeneity of variances. Logistic regression analysis was conducted to assess the relationship between each blood metal and CVD after adjusting for covariates. In the logistic model, the included covariates are: Age, Gender, BMI, Race, Education, Diabetes, and Hypertension. In which, the variable number of Age (age) in the database is RIDAGEYR. The variable number of Gender, which clearly defined as male or female is RIAGENDR. The variable number of Body mass index (BMI, kg/m^2^) is BMXBMI. The variable number of race/ethnicity which includes Mexican American, other Hispanic, non-Hispanic white, non-Hispanic black, and other races or mixed races is RIDRETH1. The variable number of education level which divided into education level below senior high school and education level above senior high school is DMDEDUC2. The variable number of Hypertension is BPQ020. The variable number of Diabetes is DID040. Random forest analysis was performed using the blood metal with or without examination variables (BMI, age and cholesterol) using R and 10-fold cross-validation. We employed a rigorous method to organize and analyze death data and clinical outcomes. The variable were UCOD LEADING, MORTSTAT, and PERMTH INT, which facilitated the accurate identification and categorization of deaths and their underlying causes. The website that can be visited is: https://www.cdc.gov/nchs/data-linkage/mortality.htm. Kaplan-Meier curve, Receiver Operating Characteristic (ROC) curves and the area under curves (AUCs) were constructed using the pROC package in *R*. Random forest model utilized by Machine learning was used to calculate the AUC score (area under the curve) and explore the potentially important blood metals associated with CVDs. In all analyses, statistical significance was considered at a *P*-value of < 0.05.

## Results

### Descriptive statistics

**Figure 1:**
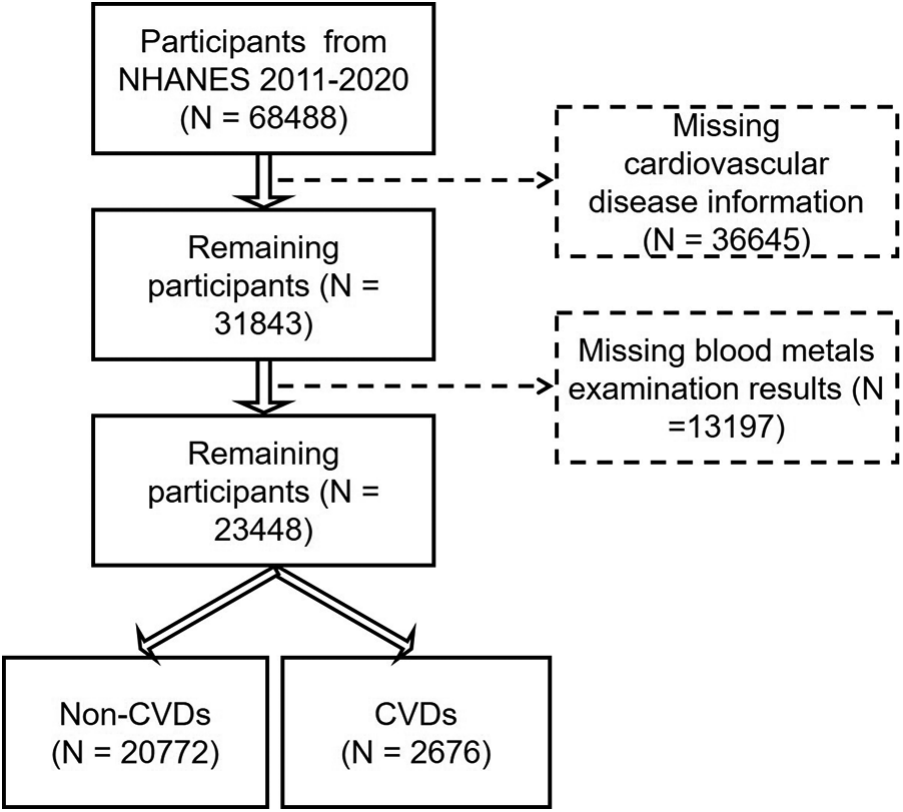
The flow diagram of study populations and the participants for the association between heavy metals and CVDs from NHANES 2011–2020.

There were 68,488 participants enrolled from NHANES 2011–2020 in the present analysis (Figure 1). In this study, CVD patients were defined as individuals with coronary heart disease, angina, congestive heart failure, stroke, or heart attack. First, we excluded participants without the information on cardiovascular disease (*n* = 36,645). Then, we excluded participants without examination of blood metals (*n* = 13,197). Finally, a total of 23,448 individuals were enrolled, including 20,772 non-CVD participants and 2,676 CVD patients.

### Demography characteristics of study participants

The demographic characteristics of the present study are shown in Table 1. A total of 23,448 participants were included in the study, with a mean age of 50.32 ± 17.59 years old and 48.5% males. We found that the prevalence of coronary heart disease, congestive heart failure, heart attack, angina, and stroke was 4.1% (*n* = 963), 3.5% (*n* = 807), 4.3% (*n* = 1,016), 2.5% (*n* = 584), and 4.5% (*n* = 1,048), respectively. In addition, the overall prevalence of total cardiovascular disease was 11.4% (*n* = 2,676), consistent with previous findings in other countries (9). Most of the subjects were Non-Hispanic White and had a high level of education. Compared to participants in the non-CVD group, those in CVD group had higher levels of blood lead (0.045 μmol/L vs. 0.610 μmol/L, *P* < 0.001), and blood cadmium (2.687 nmol/L vs. 3.559 nmol/L, *P* < 0.001). Moreover, CVD group was likely to have lower levels of blood mercury (3.700 nmol/L vs. 3.240 nmol/L, *P* < 0.001), blood selenium (2.400 μmol/L vs. 2.340 μmol/L, *P* < 0.001) and blood manganese (169.260 nmol/L vs. 157.630 nmol/L, *P* < 0.001).

**Table 1:** Characteristics of the study population.

	Overall	Non-CVDs	CVDs	*P* value
*N* (%)	23,448	20,772	2,676	
Age, years, Mean ± SD	50.32 ± 17.59	48.30 ± 17.12	66.00 ± 12.60	<0.001^b^
BMI, kg/m^2^, Mean ± SD	29.58 ± 7.29	29.44 ± 7.25	30.72 ± 7.46	<0.001^b^
Waist, cm, Mean ± SD	100.12 ± 16.77	99.42 ± 16.70	106.00 ± 16.22	<0.001^b^
Gender (%)				<0.001^a^
Male	11,375 (48.5%)	9,861 (47.5%)	1,514 (56.6%)	
Female	12,073 (51.5%)	10,911 (52.5%)	1,162 (43.4%)	
Race/ethnicity (%)				
Mexican American	2,962 (12.6%)	2,749 (13.2%)	213 (8.0%)	<0.001^a^
Other Hispanic	2,445 (10.4%)	2,218 (10.7%)	227 (8.5%)	
Non-Hispanic White	8,501 (36.3%)	7,210 (34.7%)	1,291 (48.2%)	
Non-Hispanic black	5,592 (23.8%)	4,931 (23.7%)	661 (24.7%)	
Other Races or Multi-racial	3,948 (16.8%)	3,664 (17.6%)	284 (10.6%)	
Education (%)				
<High school	4,875 (20.8%)	4,137 (19.9%)	738 (27.6%)	<0.001^a^
High school	5,391 (23.0%)	4,676 (22.5%)	715 (26.8%)	
>High school	13,154 (56.1%)	11,936 (57.5%)	1,218 (45.6%)	
Missing	28	23	5	
Current smoking status (%)				
Smoking	4,380 (18.7%)	3,826 (46.0%)	554 (34.9%)	<0.001^a^
Non-smoking	5,532 (23.6%)	4,499 (54.0%)	1,033 (65.1%)	
Missing	13,536	12,447	1,089	
Diabetes (%)	3,433 (14.6%)	2,449 (12.1%)	984 (38.3%)	<0.001^a^
Hypertension (%)	8,829 (37.7%)	6,824 (32.9%)	2,005 (75.1%)	<0.001^a^
Lead (umol/L)	0.048 (0.03, 0.075)	0.045 (0.029, 0.072)	0.610 (0.040, 0.950)	<0.001^c^
Cadmium (nmol/L)	2.760 (1.690, 5.070)	2.687 (1.601, 4.890)	3.559 (2.140, 6.317)	<0.001^c^
Mercury (nmol/L)	3.640 (1.850, 7.800)	3.700 (1.900, 7.980)	3.240 (1.700, 6.730)	<0.001^c^
Selenium (umol/L)	2.390 (2.200, 2.600)	2.400 (2.210, 2.600)	2.340 (2.140, 2.570)	<0.001^c^
Manganese (nmol/L)	167.990 (134.150, 211.120)	169.260 (135.790, 212.600)	157.630 (123.960, 198.340)	<0.001^c^

CVDs, cardiovascular diseases; BMI, Body mass index.

Data are presented as *N*% (*χ*^2^ test), Mean ± SD (independent *t*-test), Median (interquartile range) which are denoted by ^a^, ^b^ and ^c^, respectively.

### Association between blood metals and CVDs

Table 2 represents the crude and adjusted odds ratios (ORs) for the association between blood metals and CVDs. Amazingly, the crude model indicates a statistically significant difference between increased odds of CVDs and higher blood lead levels [OR (95% CI) = 13.545 (8.470–21.662) *P* < 0.001]. Futrhermore, participants with higher blood cadmium levels exhibited a higher OR for CVDs [OR (95% CI) = 1.029 (1.022–1.035) *P* < 0.001]. However, blood mercury, blood selenium, and blood manganese demonstrated an opposite trend. Notably, higher blood selenium appears to be a protective factor against CVDs, associated with a lower OR for CVDs [OR (95% CI) = 0.650 (0.573–0.737) *P* < 0.001]. Surprisingly, there was also a significant difference between increased odds of CVDs and lower blood mercury levels [OR (95% CI) = 0.991 (0.987–0.995) *P* < 0.001], traditionally considered an important environmental pollutants. Additionally, individuals with higher levels of blood manganese had lower odds of CVDs [OR (95% CI) = 0.997 (0.996–0.997) *P* < 0.001].

**Table 2:** Logistic regression analysis of blood metals on CVDs.

	OR	95% CI	*P* value
Crude model			
Lead (umol/L)	13.545	8.470–21.662	<0.001
Cadmium (nmol/L)	1.029	1.022–1.035	<0.001
Mercury (nmol/L)	0.991	0.987–0.995	<0.001
Selenium (umol/L)	0.650	0.573–0.737	<0.001
Manganese (nmol/L)	0.997	0.996–0.997	<0.001
Adjudged model			
Lead (umol/L)	1.543	0.836–2.849	0.165
Cadmium (nmol/L)	1.041	1.032–1.049	<0.001
Mercury (nmol/L)	0.995	0.990–0.999	<0.05
Selenium (umol/L)	0.786	0.688–0.898	<0.001
Manganese (nmol/L)	1.000	0.999–1.000	0.365
Gender			<0.001
Female	Ref.	Ref.	
Male	1.527	1.387–1.682	<0.001
Age, years	1.059	1.055–1.063	<0.001
BMI, kg/m^2^	1.020	1.013–1.027	<0.001
Race			<0.001
Mexican American	0.845	0.678–1.054	0.134
Other Hispanic	1.055	0.853–1.305	0.621
Non-Hispanic White	1.453	1.233–1.712	<0.001
Non-Hispanic black	1.056	0.883–1.262	0.551
Other Races or Multi-racial	Ref.	Ref.	
Education			<0.001
<High school	Ref.	Ref.	
High school	0.921	0.806–1.052	0.226
>High school	0.740	0.656–0.835	<0.001
Diabetes			<0.001
Yes	2.063	1.858–2.291	<0.001
No	Ref.	Ref.	
Hypertension			<0.001
Yes	2.737	2.461–3.045	<0.001
No	Ref.	Ref.	

After adjusting for gender, age, BMI, race, and educational background, the significance of blood lead and blood manganese disappeared. However, the relationship persisted in the adjusted model for blood cadmium, with higher levels showing a higher adjusted OR for CVDs [OR (95% CI) = 1.041 (1.032–1.049) *P* < 0.001]. Conversely, higher blood mercury levels were associated with a lower adjusted OR for CVDs [OR (95% CI) = 0.995 (0.900–0.999) *P* < 0.05]. Additionlly, the association between CVDs and blood selenium and CVDs remained significant in the adjusted model, with a lower OR for CVDs [OR (95% CI) = 0.786 (0.688–0.898) *P* < 0.001].

### Random forest modeling of CVDs using traditional risk factor and blood metals

**Figure 2:**
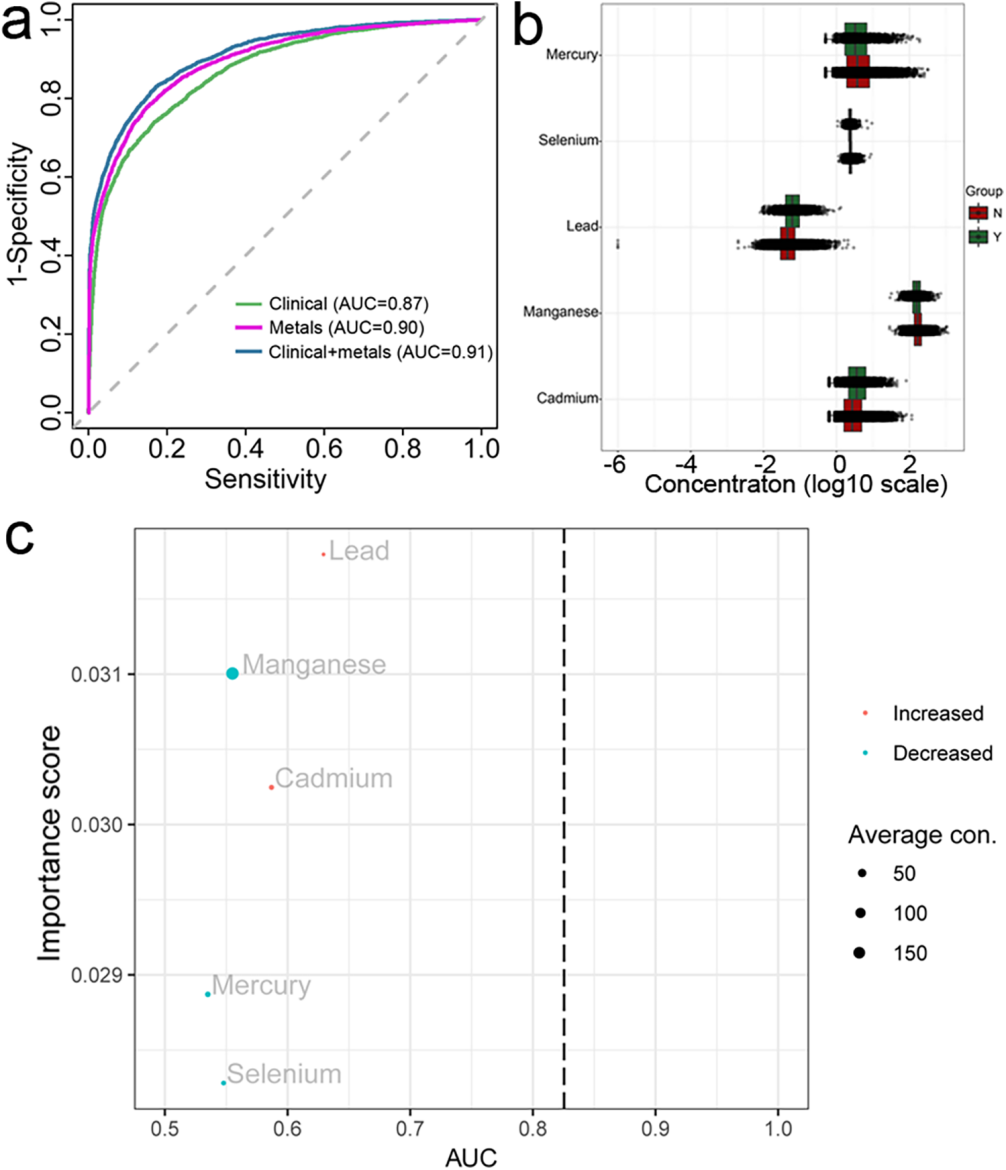
Random forest analysis and AUC curves of machine learning **(a)** presented the AUC curves performed in three models, the scatter-boxplot **(b)** presented the differences of each metal between CVDs and non-CVDs, **(c)** presented the importance score and AUC value of each metal, both B and C were calculated based on the metal-single model.

To further explore the relationship between CVDs and blood metals, we subsequently constructed prediction models for CVDs. Random forest analysis distinguished CVD patients from non-CVD individuals with an AUC of 0.87 using the clinical examination (BMI, age, and blood cholesterol) and 0.9 using blood metals, respectively (Figure 2a). Importantly, the AUC score increased to 0.91 when clinical indices and blood metals were combined. These results suggested that the blood metals have a significant predivtive effect (Figure 2a). Additionally, blood lead and blood cadmium levels were elevated in the CVDs group, whereas blood manganese, mercury, and selenium levels were decreased (Figure 2b). Consequently, the key blood metal indices included in constructing the random forest model were blood lead, manganese, cadmium, selenium, and mercury (Figure 2c). Blood lead emerged as the most important indicator in the random forest model, aligning with the findings from the logistic model.

### Association between each blood metals

**Figure 3:**
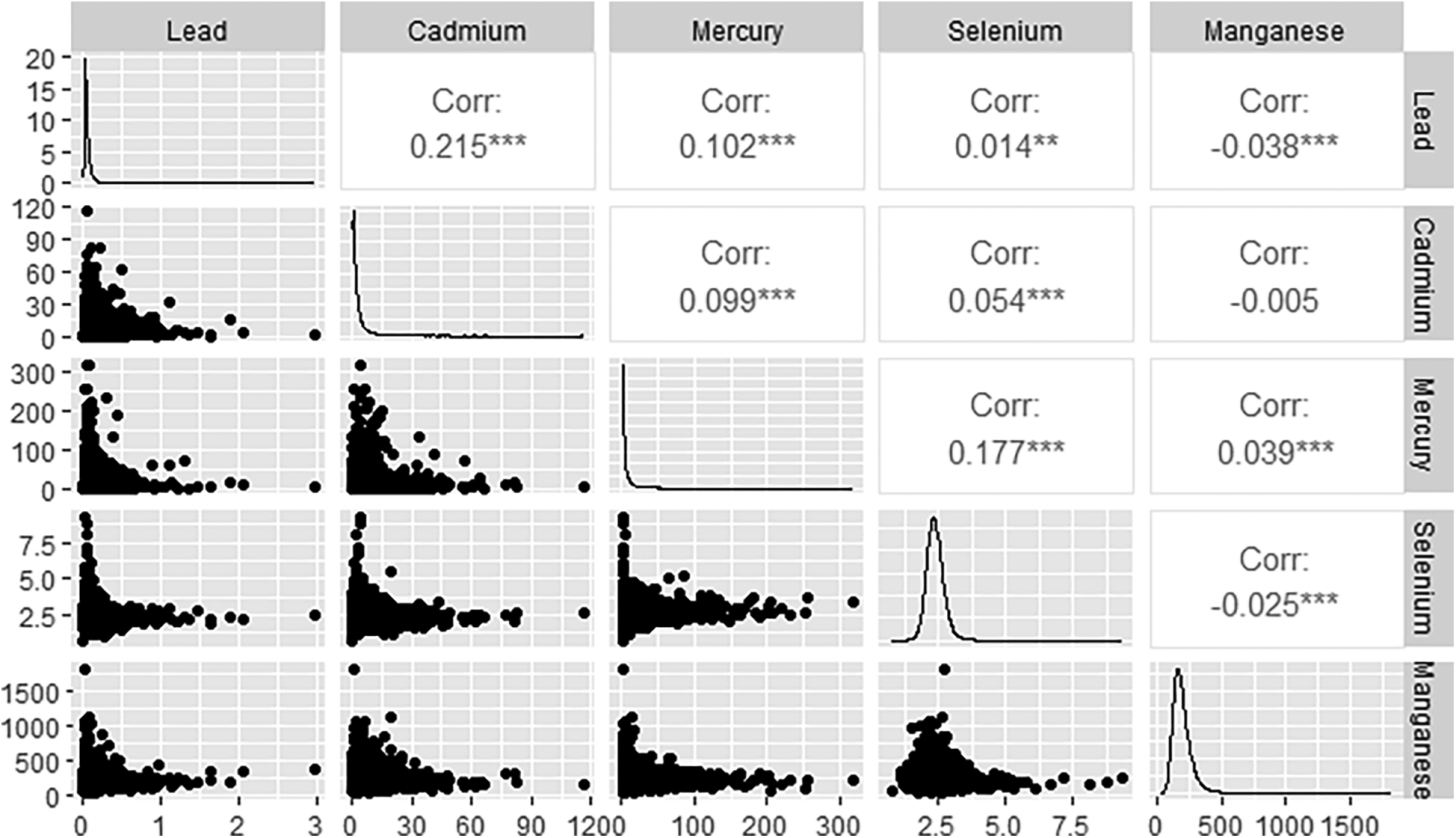
Correlate plots presented the correlation relationships between each two metals.

Figure 3 displays the associations among the various blood metals. Blood lead and blood cadmium demonstrate the strongest correlation, with an *R* value of 0.215. Additionally, the correlations between blood lead and manganese, blood cadmium and manganese, as well as blood selenium and manganese, are all negative, according to Spearman analysis.

### Association between each blood metals

**Figure 4:**
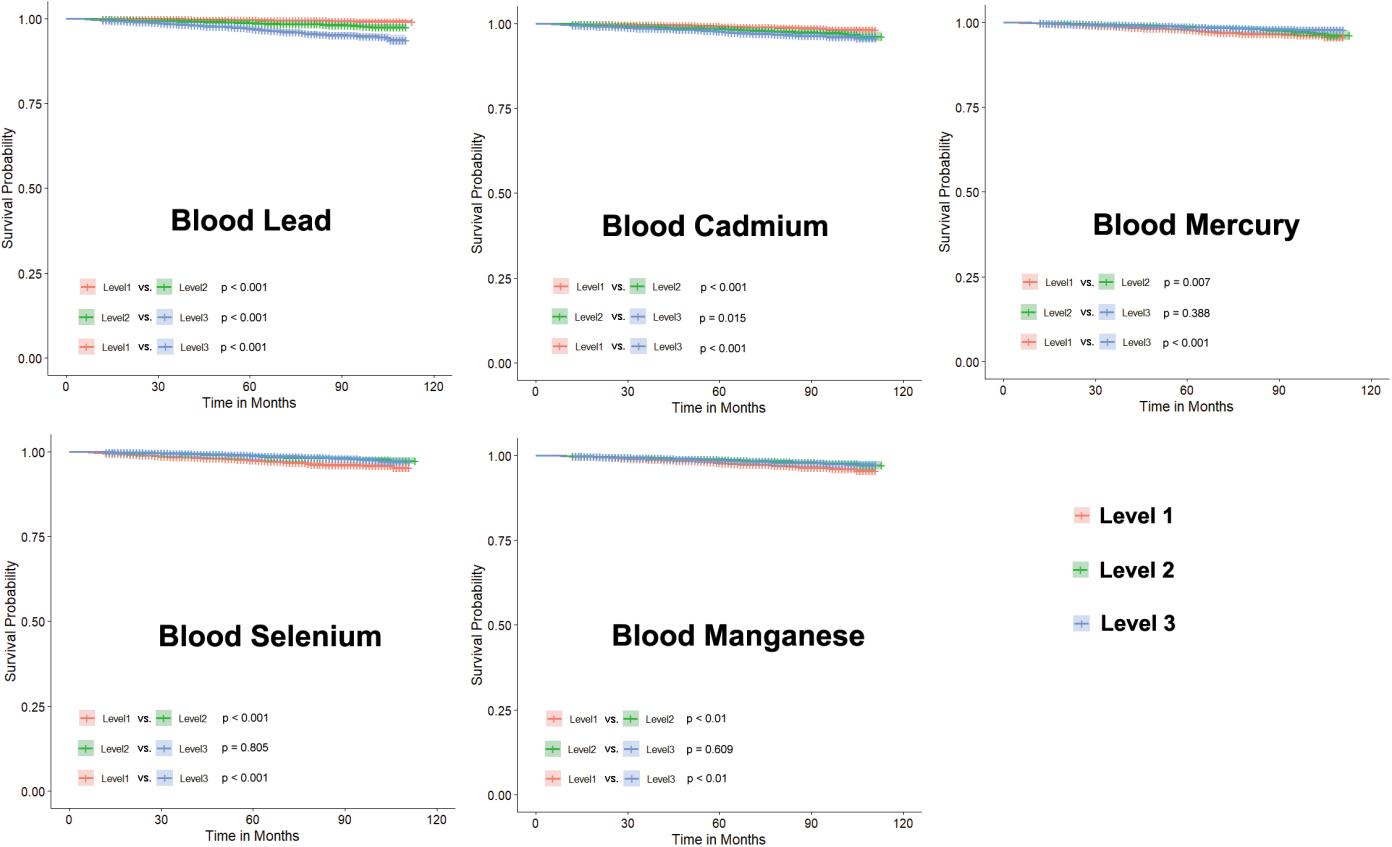
Survival analysis on participants based on tertiles of blood heavy metal levels.

We divided the levels of heavy metals in blood samples into three groups based on tertiles, labeled as Level 1, Level 2, and Level 3 according to their low, medium, and high concentrations, respectively. The concentrations of blood heavy metals in each groups is showed in Table 3. While the differences between Level 1, Level 2, and Level 3 are showed in Figure 4. These results suggest that higher levels of blood lead or cadmium are associated with a higher risk of CVD mortality.

**Table 3:** Tertiles of blood heavy metals levels.

	Level 1 (Low)	Level 2 (Middle)	Level 3 (High)	*P*-value
Lead (umol/L)	0.0247 ± 0.0073	0.0492 ± 0.0079	0.1217 ± 0.1099	<0.001
Cadmium (nmol/L)	1.3202 ± 0.4158	2.8635 ± 0.5748	9.1774 ± 6.8688	<0.001
Mercury (nmol/L)	1.5152 ± 0.5165	4.0134 ± 1.0727	18.2252 ± 19.4740	<0.001
Selenium (umol/L)	2.1311 ± 0.1432	2.4352 ± 0.0747	2.8165 ± 0.3094	<0.001
Manganese (nmol/L)	119.5281 ± 19.8824	170.1315 ± 13.8579	257.0605 ± 68.2579	<0.001

Data are presented as Mean ± SD.

## Discussion

In this study, we found that blood metals were associated with CVDs in the representative American population. Blood metals play a crucial role in the development of predicting models for CVDs. These results indivate that blood metals are an important factor that should not be overlooked in the progression of CVDs. Indeed, the frequency of accidental heavy metal pollution events has increased in the last few decades (10). Due to the adverse effects of heavy metal pollution, heavy metal-related health disorders are rapidly emerging as a significant public health problem. There is growing evidence that heavy metals may adversely affect cardiovascular health.

Mercury, lead, and cadmium are a few common pollutants that become ubiquitous in the living environment with social and economic development. Exposure to lead primarily occurs through cigarette smoking, oil paint, lead batteries, cosmetics, or contaminated food (6). Indeed, a significant number of participants from NHANES declined to respond to the questionnaire survey on smoking, potentially introducing bias. Consequently, we did not consider smoking as a factor in our analysis. Given the significant missing data on smoking, we excluded smoking as a variable when performing logistic regression modeling. Previous studies have suggested that blood lead is associated with the thickness of carotid atherosclerotic plaque (11). In addition, high blood lead is linked to coronary artery stenosis (12). Another study also indicates a correlation between lead and heart rate variability, an indicator of an abnormal cardiovascular system (13). Consistent with these previous studies, we found that blood lead levels are significantly associated with increased odds of CVDs in crude models. Furthermore, blood lead might be adverse to CVDs from the findings of the random forest model, where it also has the greatest contribution to in terms of our study. Therefore, blood lead is indeed a risk factor for CVDs. Previous findings suggest that lead is positively associated with increased levels of soluble adhesion molecules, which might be followed by an induction of oxidative stress and inflammation (14). Additionally, exposure to lead has been demonstrated to increase the release of cytokines and inflammatory factors. Some studies indicated that long-term exposure to lead influences the metabolism of lipids. Importantly, lead exhibits an effect on epigenetics from a microcosmic aspect (15). It is noteworthy that the decrease in cardiac death can be attributed to the reduction of blood lead (1). All these findings support the fact that lead is harmful to the cardiovascular system.

Furthermore, blood cadmium levels were correlated with increased odds of CVDs in the crude model. We also found that blood cadmium is a risk factor for CVD after adjusting for gender, age, BMI, race, and educational background. Blood cadmium levels have been linked to an increased risk of atherosclerosis, stroke, and heart failure (16–18). The major sources of blood cadmium include nickel-cadmium batteries, ceramic ware, and pigment (19). Similar to lead, cadmium can be absorbed through the digestive and respiratory systems, and finally deposited in the skeleton, liver, or kidneys for decades. Cadmium binds to sulfhydryl groups in the human body, leading to the disruption of glutathione and superoxide dismutase pathways, and ultimately resluting in mitochondrial or endoplasmic reticulum stress (20–22). Previous studies have indicated that high levels of urinary cadmium are associated with an increased risk of heart failure, and this association appears to be stronger in male samples (23). Additionally, patients with peripheral arterial disease patients have higher levels of urinary cadmium (24). Therefore, caution should be exercised regarding the contamination of environmental cadmium.

Another intriging finding in this study is that blood mercury is negatively correlated with CVDs in both unadjusted and adjusted models. However, the odds ratio of 0.994 suggests that this association may be a false positive or influenced by a bias. Alternatively, it could be that mercury acts as a double-edged sword, with the concentration of blood mercury potentially being maintained at low levels in these populations. Traditionally, mercury has been recognized as a risk factor for CVDs, and numerous studies have confirmed its adverse effects (25, 26).

Additionally, higher blood selenium levels appear to serve as a protective factor against cardiovascular diseases (CVDs), exhibiting a lower odds ratio in our study, which aligns with previous research findings. Selenoproteins, proteins that contain a fixed number of selenium atoms primarily in the form of the amino acid selenocysteine (Sec), play pivotal biochemical roles in the human body and constitute the primary form through which selenium exerts its biological activity in animals (27). Tommaso Angelone and colleagues demonstrated the crucial role of selenoproteins as antioxidants in cardiovascular pathophysiology, highlighting their vital importance for maintaining redox balance, antioxidant activity, as well as calcium and endoplasmic reticulum (ER) homeostasis (28).

Another study suggests a significant negative correlation between selenium intake and both CVD and all-cause mortality, hinting at selenium's potential role in preventing CVD and reducing overall mortality (29). However, some results from randomized controlled trials (RCTs) draw a different conclusion. Firstly, the relationship between selenium status and CVDs is intricate and subject to considerable debate. Secondly, several prospective observational studies indicate an association between lower selenium levels and an increased risk of CVDs. Nevertheless, the findings of RCTs are inconsistent, with some showing no preventive effect of selenium supplementation on CVDs and even suggesting a potential increase in certain risks. Furthermore, the results of meta-analyses indicate that the association between selenium status and CVDs may be influenced by various factors, including study design, sample size, selenium measurement methodologies, and definitions of CVD (30). Therefore, more in-depth research is needed to fully understand the complex relationship between selenium, selenoproteins, and CVDs.

Another important blood metal that contributes to the prediction model for CVDs is manganese. Blood manganese exhibited an opposite trend compared with blood lead and cadmium, appearing to be a protective factor for CVDs. Our previous study refers to dietary inflammation and suggests that dietary magnesium is beneficial for heart failure (31).

However, there are still some limitations of the present study. First, our study is a cross-sectional study, which may not provide definitive causal relationships. Further investigation, exploring mechanisms through animal experiments, is also required. Additionally, the blood metal elements analyzed in our study are based on a single time point; having multiple time point analyses could potentially make the conclusions more reliable. Furthermore, due to the numerous metal elements present in blood, we only analyzed five of them. Whether there are synergistic effects involving other metals also requires further investigation.

In conclusion, our present study suggests that a mixture of blood metals is associated with the risk of CVDs. The heavy metals that may increase the risk of CVDs are lead and cadmium. Management and control the heavy metal pollution might reduce the incidence of CVDs.

## Data Availability

The raw data supporting the conclusions of this article will be made available by the authors, without undue reservation.

## References

[1] Ruiz-Hernandez A Navas-Acien A Pastor-Barriuso R Crainiceanu CM Redon J Guallar E Declining exposures to lead and cadmium contribute to explaining the reduction of cardiovascular mortality in the US population, 1988–2004. Int J Epidemiol. (2017) 46:1903–12. 10.1093/ije/dyx176 29025072 PMC5837785

[2] Cena H Calder PC. Defining a healthy diet: evidence for the role of contemporary dietary patterns in health and disease. Nutrients. (2020) 12:334. 10.3390/nu12020334 32012681 PMC7071223

[3] Glovaci D Fan W Wong ND. Epidemiology of diabetes Mellitus and cardiovascular disease. Curr Cardiol Rep. (2019) 21:21. 10.1007/s11886-019-1107-y 30828746

[4] Bourdrel T Bind MA Béjot Y Morel O Argacha JF. Cardiovascular effects of air pollution. Arch Cardiovasc Dis. (2017) 110:634–42. 10.1016/j.acvd.2017.05.003 28735838 PMC5963518

[5] Guo X Li N Wang H Su W Song Q Liang Q Combined exposure to multiple metals on cardiovascular disease in NHANES under five statistical models. Environ Res. (2022) 215:114435. 10.1016/j.envres.2022.114435 36174761

[6] Lamas GA Bhatnagar A Jones MR Mann KK Nasir K Tellez-Plaza M Contaminant metals as cardiovascular risk factors: a scientific statement from the American Heart Association. J Am Heart Assoc. (2023) 12:e29852. 10.1161/JAHA.123.029852 PMC10356104 37306302

[7] Tchounwou PB Yedjou CG Patlolla AK Sutton DJ. Heavy metal toxicity and the environment. Exp Suppl. (2012) 101:133–64. 10.1007/978-3-7643-8340-4_6 22945569 PMC4144270

[8] Lamas GA Navas-Acien A Mark DB Lee KL. Heavy metals, cardiovascular disease, and the unexpected benefits of chelation therapy. J Am Coll Cardiol. (2016) 67:2411–8. 10.1016/j.jacc.2016.02.066 27199065 PMC4876980

[9] Dornquast C Kroll LE Neuhauser HK Willich SN Reinhold T Busch MA. Regional differences in the prevalence of cardiovascular disease. Dtsch Arztebl Int. (2016) 113:704–11. 10.3238/arztebl.2016.0704 27866565 PMC5143789

[10] Yuan Y Xiang M Liu C Theng B. Chronic impact of an accidental wastewater spill from a smelter, China: a study of health risk of heavy metal(loid)s via vegetable intake. Ecotoxicol Environ Saf. (2019) 182:109401. 10.1016/j.ecoenv.2019.109401 31272024

[11] Harari F Barregard L Östling G Sallsten G Hedblad B Forsgard N Blood lead levels and risk of atherosclerosis in the carotid artery: results from a Swedish cohort. Environ Health Perspect. (2019) 127:127002. 10.1289/EHP5057 31808705 PMC6957277

[12] Kim S Kang W Cho S Lim DY Yoo Y Park RJ Associations between blood lead levels and coronary artery stenosis measured using coronary computed tomography angiography. Environ Health Perspect. (2021) 129:27006. 10.1289/EHP7351 33621129 PMC7901725

[13] Yu CG Wei FF Yang WY Zhang ZY Mujaj B Thijs L Heart rate variability and peripheral nerve conduction velocity in relation to blood lead in newly hired lead workers. Occup Environ Med. (2019) 76:382–8. 10.1136/oemed-2018-105379 30928907 PMC6585574

[14] Camaj PR Graziano JH Preteni E Popovac D LoIacono N Balac O Long-Term effects of environmental lead exposure on blood pressure and plasma soluble cell adhesion molecules in young adults: a follow-up study of a prospective cohort in Kosovo. J Environ Public Health. (2018) 2018:3180487. 10.1155/2018/3180487 29535789 PMC5817317

[15] Mitra P Sharma S Purohit P Sharma P. Clinical and molecular aspects of lead toxicity: an update. Crit Rev Clin Lab Sci. (2017) 54:506–28. 10.1080/10408363.2017.1408562 29214886

[16] Mao Q Zhou D Sun Y Zhao J Xu S Zhao X. Independent association of blood cadmium with subclinical lower extremity atherosclerosis: an observational study based on dose-response analysis. Chemosphere. (2023) 313:137441. 10.1016/j.chemosphere.2022.137441 36470359

[17] Borné Y Fagerberg B Persson M Östling G Söderholm M Hedblad B Cadmium, carotid atherosclerosis, and incidence of ischemic stroke. J Am Heart Assoc. (2017) 6(12):e006415. 10.1161/JAHA.117.006415 PMC5778998 29197829

[18] Xing X Xu M Yang L Shao C Wang Y Qi M Association of selenium and cadmium with heart failure and mortality based on the national health and nutrition examination survey. J Hum Nutr Diet. (2023) 36:1496–506. 10.1111/jhn.13107 36321401

[19] Järup L. Hazards of heavy metal contamination. Br Med Bull. (2003) 68:167–82. 10.1093/bmb/ldg032 14757716

[20] Hormozi M Mirzaei R Nakhaee A Izadi S Dehghan HJ. The biochemical effects of occupational exposure to lead and cadmium on markers of oxidative stress and antioxidant enzymes activity in the blood of glazers in tile industry. Toxicol Ind Health. (2018) 34:459–67. 10.1177/0748233718769526 29669482

[21] Chen J Pan T Wan N Sun Z Zhang Z Li S. Cadmium-induced endoplasmic reticulum stress in chicken neutrophils is alleviated by selenium. J Inorg Biochem. (2017) 170:169–77. 10.1016/j.jinorgbio.2017.02.022 28249225

[22] Omar UM Elmorsy EM Al-Ghafari AB. Mitochondrial disruption in isolated human monocytes: an underlying mechanism for cadmium-induced immunotoxicity. J Immunotoxicol. (2022) 19:81–92. 10.1080/1547691X.2022.2113840 36067115

[23] Borné Y Barregard L Persson M Hedblad B Fagerberg B Engström G. Cadmium exposure and incidence of heart failure and atrial fibrillation: a population-based prospective cohort study. BMJ Open. (2015) 5:e7366. 10.1136/bmjopen-2014-007366 PMC4480021 26078311

[24] Tellez-Plaza M Guallar E Fabsitz RR Howard BV Umans JG Francesconi KA Cadmium exposure and incident peripheral arterial disease. Circ Cardiovasc Qual Outcomes. (2013) 6:626–33. 10.1161/CIRCOUTCOMES.112.000134 24255048 PMC4190067

[25] Sun Y Liu B Rong S Zhang J Du Y Xu G Association of seafood consumption and mercury exposure with cardiovascular and all-cause mortality among US adults. JAMA Netw Open. (2021) 4:e2136367. 10.1001/jamanetworkopen.2021.36367 34842923 PMC8630568

[26] Hu XF Lowe M Chan HM. Mercury exposure, cardiovascular disease, and mortality: a systematic review and dose-response meta-analysis. Environ Res. (2021) 193:110538. 10.1016/j.envres.2020.110538 33285155

[27] Rocca C Pasqua T Boukhzar L Anouar Y Angelone T. Progress in the emerging role of selenoproteins in cardiovascular disease: focus on endoplasmic reticulum-resident selenoproteins. Cell Mol Life Sci. (2019) 76:3969–85. 10.1007/s00018-019-03195-1 31218451 PMC11105271

[28] Angelone T Rocca C Lionetti V Penna C Pagliaro P. Expanding the frontiers of guardian antioxidant selenoproteins in cardiovascular pathophysiology. Antioxid Redox Signal. (2024) 40:369–432. 10.1089/ars.2023.0285 38299513

[29] Jenkins D Kitts D Giovannucci EL Sahye-Pudaruth S Paquette M Blanco MS Selenium, antioxidants, cardiovascular disease, and all-cause mortality: a systematic review and meta-analysis of randomized controlled trials. Am J Clin Nutr. (2020) 112:1642–52. 10.1093/ajcn/nqaa245 33053149 PMC7727482

[30] Zhang X Liu C Guo J Song Y. Selenium status and cardiovascular diseases: meta-analysis of prospective observational studies and randomized controlled trials. Eur J Clin Nutr. (2016) 70:162–9. 10.1038/ejcn.2015.78 25990689

[31] Liu Z Liu H Deng Q Sun C He W Zheng W Association between dietary inflammatory Index and heart failure: results from NHANES (1999–2018). Front Cardiovasc Med. (2021) 8:702489. 10.3389/fcvm.2021.702489 34307508 PMC8292138

